# The Effect of Acute Aerobic Exercise On the Time Spent in Hypoglycaemia After Bariatric Surgery (The BariEX Study)

**DOI:** 10.1007/s11695-026-08712-3

**Published:** 2026-05-01

**Authors:** Israel Podesta-Donoso, Dimitris Papamargaritis, Joe Henson, Stanislava Katsarova-Harrison, Aikaterina Tziannou, Helen Waller, Alex V Rowlands, David Bowrey, Oluwaseun Anyiam, Iskandar Idris, David J Stensel, David R Webb, Anjali Zalin, Tom Yates, Pratik Choudhary, Melanie J Davies, Louisa Y Herring

**Affiliations:** 1 Diabetes Research Centre, College of Life Sciences, University of Leicester, Leicester, United Kingdom; 2 NIHR Leicester Biomedical Research Centre, Leicester, United Kingdom; 3 National Centre for Sport and Exercise Medicine, School of Sport, Exercise and Health Sciences,, Loughborough University, Loughborough, United Kingdom; 4 Leicester Diabetes Centre, University Hospitals of Leicester NHS Trust, Leicester, United Kingdom; 5 Department of Surgery, Leicester Royal Infirmary, University Hospitals of Leicester NHS Trust, Leicester, United Kingdom; 6 Centre of Metabolism Ageing & Physiology, School of Medicine, University of Nottingham, Nottingham, United Kingdom; 7 National Centre for Sport and Exercise Medicine, School of Sport, Exercise and Health Sciences,, Loughborough University, Loughborough, United Kingdom; 8 Faculty of Sport Sciences, Waseda University, Tokyo, Japan; 9 Department of Bariatric and Upper Gastrointestinal Surgery, Luton and Dunstable University Hospital NHS Foundation Trust, Luton, United Kingdom

## Abstract

**Objective:**

To determine whether a pre-lunch single bout of moderate-intensity aerobic exercise (AEX) alters time spent in hypoglycaemia (<3.0 mmol/L) during the subsequent 24 hours (24-h) and parameters of glucose homeostasis in individuals without diabetes after metabolic/bariatric surgery (MBS).

**Methods:**

In a randomised crossover study, 15 participants completed two conditions: 30min treadmill walking at 60% V̇O₂peak (AEX) and time-matched sitting (CON). After an overnight fast and a standardised breakfast, participants performed AEX or CON, and both conditions were followed by an identical lunch administered as a mixed-meal tolerance test (MMTT). Continuous glucose monitoring (CGM) assessed glucose levels for 24-h post-intervention, during which participants consumed standardised meals. **CGM data were available for analysis in 11 participants.** The primary outcome was time spent with glucose <3.0 mmol/L during the 24-h post-intervention. Secondary outcomes included other CGM-derived glucose metrics and plasma glucose and insulin responses during the 3-h MMTT.

**Results:**

Only one isolated hypoglycaemic event (<3.0 mmol/L) occurred, precluding statistical analysis of the primary outcome. Mean 24-h glucose (AEX: 6.4 (1.0); CON: 6.5 (0.9) mmol/L; p = 0.57) and time <3.9 mmol/L (AEX 0 (0.0, 0.5); CON 0 (0.0, 0.5) %; p = 0.68) did not differ between conditions. AEX reduced glucose coefficient of variation (p < 0.01). During the MMTT, nadir, peak, and AUC_0-180_ glucose, as well as pre-MMTT insulin concentrations, were higher following AEX (all p < 0.05).

**Conclusions:**

A pre-lunch 30-min bout of AEX did not increase the 24-h risk of hypoglycaemia post-MBS but elevated post-MMTT glucose levels.

**Supplementary Information:**

The online version contains supplementary material available at 10.1007/s11695-026-08712-3.

## Key Points

A 30-minute session of moderate-intensity aerobic exercise before lunch did not increase 24-hour hypoglycaemia risk in individuals without diabetes after bariatric surgery.

Aerobic exercise reduced 24-hour glucose variability but caused an increase in postprandial glucose concentration during the subsequent mixed-meal tolerance test.

**Supplementary Information:**

The online version contains supplementary material available at 10.1007/s11695-026-08712-3.

## Introduction

Within obesity treatments, metabolic and bariatric surgery (MBS) is currently the most effective method to achieve substantial long-term weight loss (WL) in people with class II or III obesity [1]. Despite successful and sustained WL with MBS, nutritional deficiencies, dumping syndrome, and postprandial hyperinsulinaemic hypoglycaemia (PHH) are post-operative complications that can affect long-term metabolic outcomes [2].

PHH is common after MBS [3] and is defined as glucose levels < 3.0 mmol/L and/or symptoms suggestive of hypoglycaemia 1–3 h (h) after a meal [4–6]. Data from continuous glucose monitoring (CGM) shows that up to 70% of people without type 2 diabetes (T2D) who have undergone MBS spend 2–5% of their time in hypoglycaemia [3, 7]. It is well recognised that many episodes of CGM-detected hypoglycaemia are asymptomatic [7, 8], but up to 35% of people undergoing MBS experience symptoms of PHH during daily life [9]. Recurrent episodes of PHH after MBS are associated with reduced quality of life [10], postoperative weight regain [11], and may contribute to increased frequency of seizures and syncope after MBS [12].

The exact pathophysiology of PHH following MBS remains unclear. It likely involves the rapid delivery and absorption of large carbohydrate loads in the distal small intestine due to altered gastrointestinal anatomy, leading to exaggerated insulin release and increased secretion of gut hormones that regulate glucose homeostasis [6, 13–16]. The risk factors for developing PHH after MBS include younger age, absence of T2D, greater pancreatic beta cell function, and higher insulin sensitivity [9, 17]. Treatment options for PHH are limited, with dietary modification being the first-line treatment [2].

Exercise is a key adjunct therapy after MBS, offering multiple health benefits and enhancing long-term outcomes [18]. It improves cardiorespiratory fitness, quality of life, physical function, and body composition [17–21], and helps mitigate weight regain and recurrence of obesity-related complications [19, 22, 23]. European post-MBS guidelines recommend at least 30 min of moderate-intensity aerobic exercise (AEX) daily [24]. Importantly, AEX performed after MBS can increase insulin sensitivity for 24–48 h in individuals without diabetes by enhancing muscle glucose uptake [25]; however, this increased insulin responsiveness may also elevate the risk of PHH [17].

Currently, there is limited evidence on the effects of a single session of AEX on glucose levels and hypoglycaemia risk after MBS in individuals without T2D. One previous study examined the impact of 30-minute postprandial AEX on glucose and hormonal responses compared to resting conditions. This study involved participants who had undergone RYGB, with or without symptoms suggestive of PHH, after consuming either a high- or low-glycaemic index breakfast. AEX was initiated within 10 min of breakfast consumption following an overnight fast. The results showed a trend toward higher nadir plasma glucose levels with AEX for both types of breakfasts, although these findings were limited to the immediate post-meal period [26]. Research is lacking on the effects of AEX after MBS on glucose homeostasis beyond the first 4 h after eating, or when AEX is performed at other times of day. Interestingly, acute AEX performed before a second meal (e.g. lunch) has been reported to paradoxically increase postprandial glucose responses to that meal, a phenomenon referred to as the “paradoxical second-meal effect.” Acute exercise is also associated with transient increases in counter-regulatory hormones, including catecholamines and glucagon, which stimulate hepatic glucose production and may also influence glucose responses to the following meal. However, to date no studies have examined the effect of AEX performed before a second meal on postprandial glycaemia as well as the 24-h hypoglycaemia risk in individuals after MBS. Therefore, our study assessed how a single session of AEX performed before lunch affects (A) the time spent in hypoglycaemia - defined as sensor glucose levels below 3.0 mmol/L - over the subsequent 24 h in individuals without established PHH, and (B) postprandial glucose homeostasis parameters following lunch.

## Materials and Methods

Participants were recruited in this randomised crossover trial between June 2022 and September 2024 through multiple pathways - primary and secondary care, participants of previous research studies as well as from community settings.

Eligible participants included those who had undergone MBS (RYGB or SG) ≥ 12 months before entering the trial, aged ≥ 18 to < 75 years, without diabetes, who were able to understand written and spoken English and to provide informed consent. Key exclusion criteria included a known diagnosis of PHH or use of medications that could affect glucose levels. People with PHH were excluded due to uncertainty regarding whether acute AEX could exacerbate hypoglycaemia risk in this population. A full list of inclusion and exclusion criteria is available in the supplementary material. Ethical approval for the study was obtained by the London-Bromley Research Ethics Committee (ref 21/LO/004). This trial was prospectively registered on the ISRCTN registry (ISRCTN 17674908) and conducted in accordance with the Declaration of Helsinki. All study visits were conducted at the Leicester Diabetes Centre, UK. Patient and public involvement (PPI) representatives reviewed the patient-facing study information to ensure the language was clear, accessible and understandable.

### Study Design

**Fig. 1:**
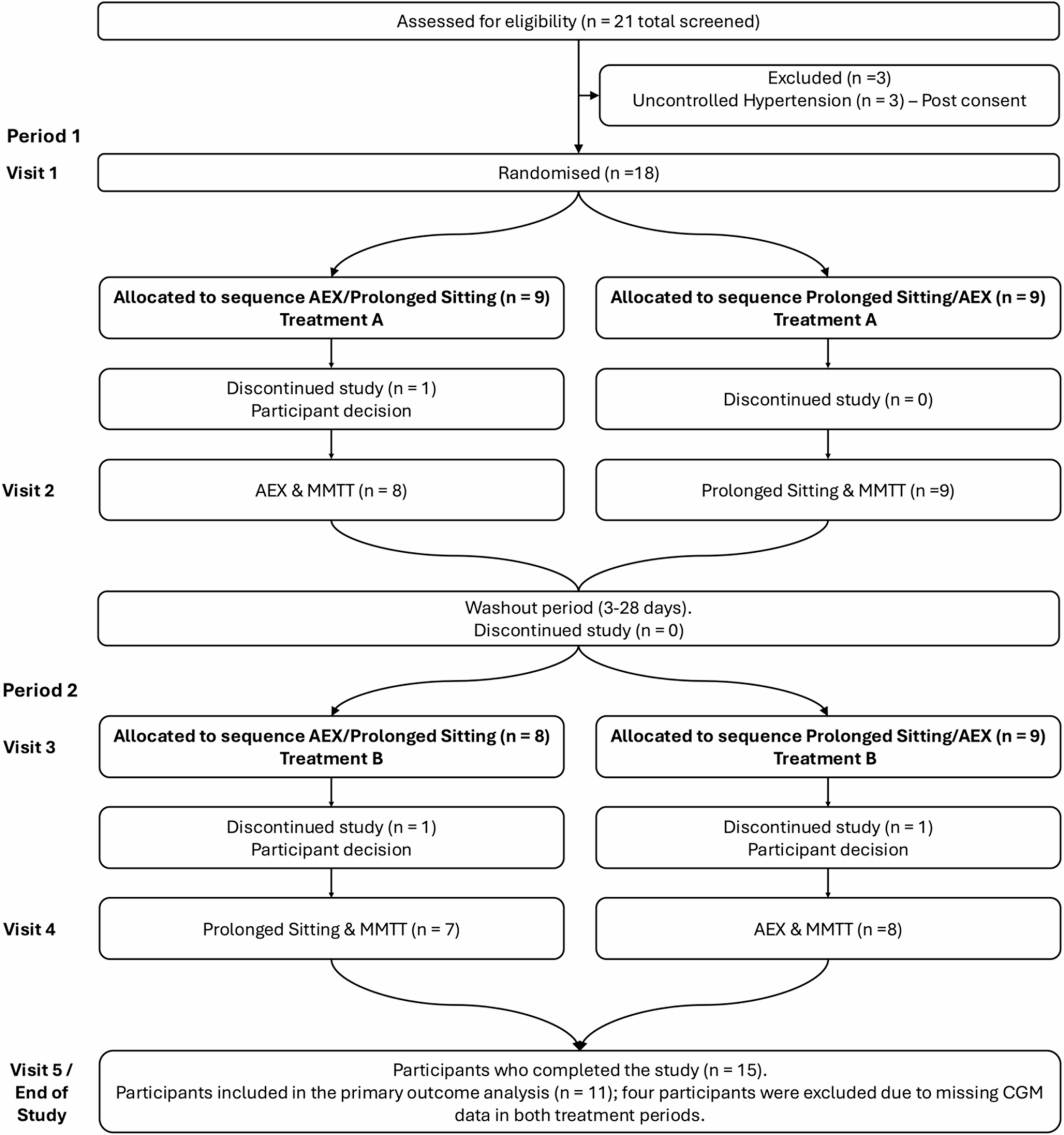
The BariEX study CONSORT diagram. AEX = Moderate-intensity Aerobic Exercise; CGM = Continuous Glucose Monitoring; MMTT = Mixed-Meal Tolerance Test

Figure 1 Outlines the study CONSORT diagram.

## Visit 0 - Screening and Eligibility Visit

After verifying eligibility and obtaining signed informed consent, a baseline assessment was performed by a trained clinician. Data on demographics, anthropometrics, medical history, and medication use were collected, followed by a blood sample and a urine pregnancy test for female participants. A maximal exercise test (See Supplementary material) was conducted to assess cardiorespiratory fitness (V̇O_2_peak) and to prescribe the exercise intensity. Participants wore a GENEActiv accelerometer (ActivInsights, Kimbolton, UK) to quantify habitual moderate-vigorous physical activity (MVPA) levels throughout the study (i.e., from visit 0 to visit 5; See supplementary material). Additionally, participants were provided with a glucose meter (Contour Plus Blue, Ascensia Diabetes Care, UK) for safety monitoring during the study. Participants were informed about symptoms suggestive of hypoglycaemia and instructed to measure capillary glucose levels only if such symptoms occurred. If hypoglycaemia was confirmed, they were advised to treat the episode according to standard hypoglycaemia management. The glucose meter was provided for safety monitoring only, and participants were not given behavioural advice to prevent hypoglycaemia.

**Fig. 2:**
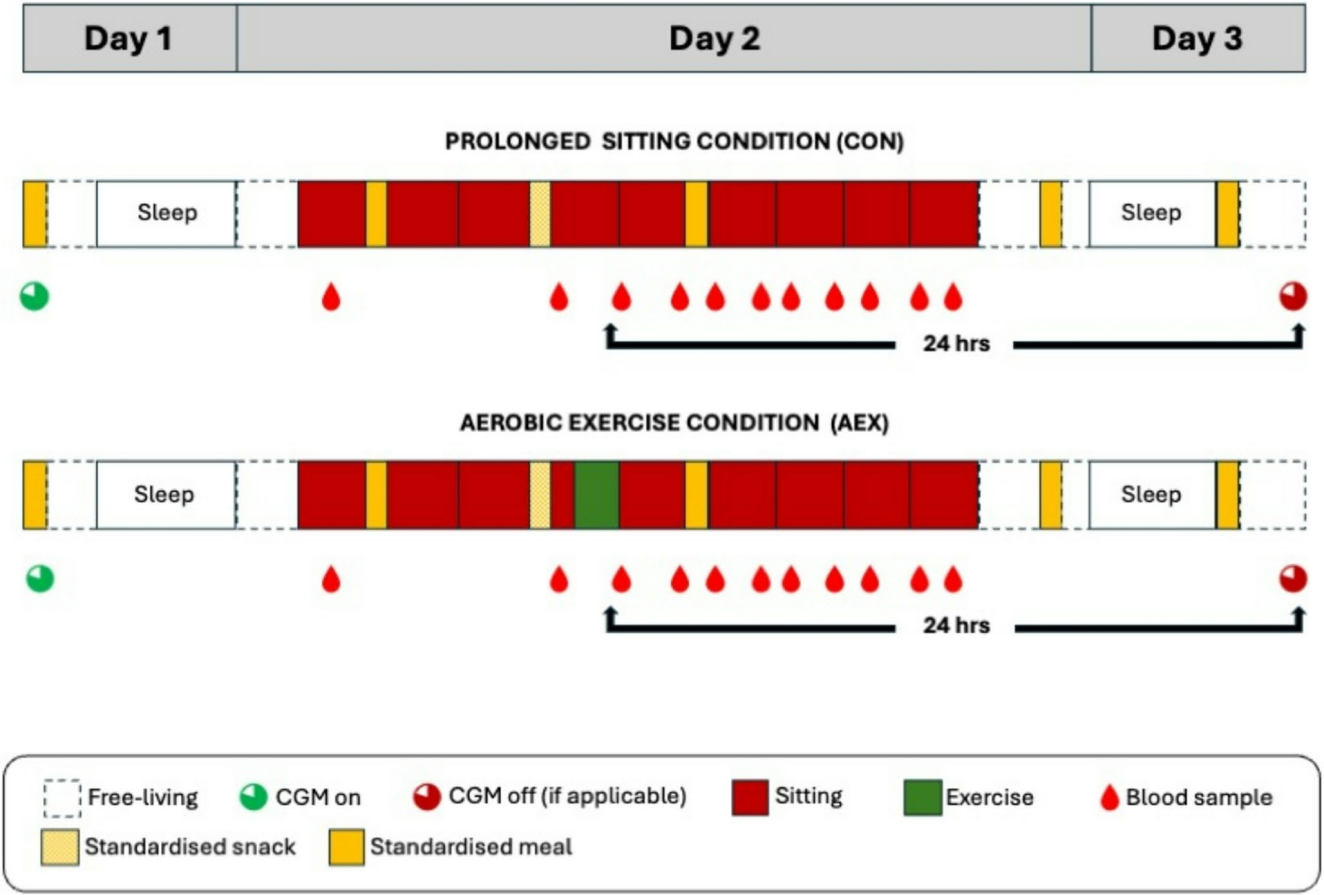
Schematic representation of each treatment period. Prolonged Sitting condition (CON) (top panel) and Aerobic Exercise (AEX) (bottom panel). CGM = Continuous Glucose monitoring

Participants who were confirmed eligible after screening and maximal exercise testing were randomly assigned (1:1) to one of two experimental sequences via an independent computer-generated allocation system (sealedenvelope.com; Fig. 2), with the sequence concealed from study personnel and released only after eligibility was confirmed:

1. Control (CON) condition followed by AEX condition (CON–AEX): Participants first underwent a CON condition consisting of approximately 6 h and 45 min of prolonged sitting. Subsequently, they completed an AEX condition, comprising approximately 6 h and 15 min of sitting interrupted by a single 30-min bout of AEX at 60% of their V̇O₂peak.
2. AEX condition followed by CON condition (AEX–CON): Participants first completed the AEX condition, as described above, followed by the CON condition.

## Treatment Period

### Day 1 on Each Treatment (Visits 1 and 3)

Participants attended the research facility to be fitted with a blinded CGM device (Dexcom G6). They were also given a 3-day food and glucose record diary, as well as a standardised evening meal to be consumed at a designated time before day 2 of each treatment period (for detailed information regarding the food provided, refer to the supplementary material).

### Day 2 (Visits 2 and 4)

Participants arrived at ~ 8:30 am after ≥ 10 h of overnight fasting and after refraining from MVPA and alcohol for at least 24 h before intervention visits. Compliance with these conditions was assessed by self-report upon arrival. Body weight, blood pressure, and heart rate were measured on arrival. A cannula was inserted, and a blood sample was collected immediately before consumption of a standardised breakfast.

After finishing breakfast, the experimental period commenced (+ 0 h). Following a 2-h rest to ensure a stable baseline, participants consumed a standardised 15 g carbohydrate snack (two shortcake biscuits, Tesco, UK; for detailed nutritional information, see the supplementary material) 15 min before the AEX intervention. This snack was also provided in the CON session to ensure nutritional consistency. Participants underwent either the AEX (treadmill walking), lasting 35 min in the exercise lab (starting at +2 h 15 min and ending at +2 h 50 min), or CON, which involved prolonged sitting for the same duration. Blood samples were taken just before (+ 2 h 15 min) and immediately after the intervention (+ 2 h 50 min).

During both conditions, participants were supervised throughout. The AEX session included a 3-min warm-up, 30-min walk at 60% V̇O₂ peak, and a 2-min cool-down. In the CON condition, participants remained seated.

After the completion of the designated intervention, approximately +3 h 30 min into the experimental period, participants underwent a mixed meal tolerance test (MMTT) as lunch [30% total daily energy intake requirements; 47% carbohydrates, 18% protein, and 35% fat (Fortisip Compact Protein, Nutricia Limited, UK & Belvita soft bake bars, Mondelez, UK)- See MMTT section below)]. During the MMTT, blood samples for measurement of glucose and insulin were taken immediately before the meal (+ 3 h 30 min), and then at 15, 30, 60, 90, 120, 150, and 180 min after finishing the meal. Once the 3-h blood sampling period was completed (≈ + 6 h 45 min), participants were allowed to leave the facilities. Participants received a standardised meal to take home and consume that evening. They were also given a standardised breakfast for the following day (day 3 of each investigation period) with instructions to eat it at a specified time and avoid other foods, ensuring dietary intake was standardised for 24 h after the intervention completion.

Throughout the intervention visits, the study clinical team monitored symptoms of hypoglycaemia. Participants were instructed to notify the study team if they experienced symptoms suggesting hypoglycaemia, at which point the study staff would measure capillary glucose levels (Contour Plus Blue, Ascensia Diabetes Care, UK). If hypoglycaemia (glucose < 3.0mmol/L at the bedside) was confirmed, treatment was provided. No participant required treatment for hypoglycaemia during the MMTT in either study condition.

### Day 3 (Conditional Visit and Visit 5)

For the first treatment period, participants returned the accelerometer, CGM and the diet diaries at visit 3 (corresponding to day 1 of the second treatment period). A minimum 3-day washout period was required between the two treatment periods, with a maximum of 28 days. On day 3 of the second treatment period (visit 5), participants had to wait at least 24 h after completion of the intervention (post-midday) before arriving at the facilities to remove the accelerometer and CGM, and return the glucose monitoring diary, the 3-day diet diary, and the glucose meter.

## Measurements During the Study

### Continuous Glucose Monitoring (CGM)

Participants used a blinded CGM device (Dexcom, G6, Dexcom, Inc., USA) for both treatment periods. Data were downloaded via Dexcom clarity and analysed using R package iglu [27]. CGM data processing and outcome calculation were performed using the closest available glucose measurement to each participant’s intervention completion as the starting time point. All available CGM measurements were then included for the subsequent 24-h period to calculate CGM-related outcomes.

### Blood Samples

#### Laboratory Tests/Biochemical Analysis (Screening, Visits 2 and 4)

Plasma glucose was measured from venous blood collected in 2.7 mL fluoride monovettes and analysed via a Glucose Hexokinase 3 assay on the Atelica CH (SIEMENS) at the University Hospitals of Leicester NHS Trust. For insulin measurement, venous blood was drawn into 4.9 mL EDTA (Ethylenediamine tetra-acetic acid) monovettes, centrifuged at 4 °C for 10 min at 1500 g, and plasma was aliquoted into Eppendorf tubes, then frozen at −20 °C and transferred to −80 °C the same day. Plasma insulin was measured using a Magnetic Luminex Assay (R&D Systems, Minneapolis, MN, USA) on a Luminex LX200 with Xponent software (Luminex Corporation, Austin, TX, USA). The mean intraplate coefficient of variation was 3.6%.

#### Mixed Meal Tolerance Test

The MMTT comprised 30% of the Mifflin-St Jeor total daily energy intake requirements with macronutrient composition as described above. For two participants, an alternative MMTT was used due to known food intolerance. The alternative MMTT was composed of yoghurt (Brooklea 10% Greek Strained Yogurt, Brooklea, Germany) and jam (Bonne Maman Strawberry, Andros group, France), with macronutrient composition of 49% carbohydrates, 11% protein and 39% fat. Participants were given 15–20 min to consume the MMTT.

#### Diet Diaries

To assess dietary intake and compliance with standardised meal provision, participants were asked to complete 3-day diet diaries. Detailed verbal and written instructions on how to complete a diet diary were provided to participants, who were asked to include all food and fluids consumed.

### Statistics

Baseline participant characteristics are reported as mean (SD) or median (quartiles) for continuous variables, and as a counts (n, %) for categorical variables. The normality of data was assessed using the Shapiro-Wilk test. The sample size calculation was based on previous research findings [3, 28]. To detect a 25% difference (18 min/day, SD = 25 min) in time spent in hypoglycaemia (defined as glucose levels during the first 24 h between the AEX and control interventions) we aimed to recruit 18 participants, accounting for a 20% drop-out rate, with a significance level set at 5% and 80% statistical power. For the analysis of primary and secondary CGM outcomes, paired t-tests or non-parametric equivalents were used as appropriate for treatment group comparisons. To ensure data quality and reliability, only participants with at least 16 h and 48 min of CGM data (representing 70% data completeness) during this 24-h post-intervention window were included.

For outcomes including plasma glucose and insulin concentration values obtained during the MMTT, linear mixed regression models were fitted, with an exchangeable correlation matrix. Fixed effects included randomisation group, visit number, pre-exercise, post-exercise and fasting values of the outcome and participant-specific random effects. For Area Under the Curve (AUC) calculations during the MMTT, the trapezoidal rule was applied. For AUC-related outcomes, mixed regression models were fitted as above, with the only difference being the addition of post-exercise values in the AUC calculation. Model residual assumptions were tested, and log-transformations were applied when needed. Missing data on insulin and glucose during the MMTT were imputed (8 out of 330 for insulin and 6 out of 330 values for glucose), provided the availability of ≥ 50% of data points within the MMTT.

The collected diet diaries were input and analysed by the study dietitian using the nutritional analysis software Nutritics (Research Edition, v5.09, Dublin, Nutritics, 2019). Total daily energy intake was estimated in kJ/day, and macronutrients (carbohydrates, protein, fat) were estimated in g/day. For all participants included in the diet diaries analysis (*n* = 14), paired sample t-tests were conducted to compare dietary intake 24 h after the start of each intervention between the two treatment periods.

Analyses for primary and secondary outcomes were carried out in R version 4.4.2. Significance level was set to 5% unless otherwise stated.

## Results

### Participants Characteristics

Baseline characteristics of participants who were randomised, completed the intervention, and were included in the primary outcome analysis are detailed in Table 1. The characteristics of RYGB participants are shown in Supplementary Table 1. The median washout period between treatment periods was 5 days (IQR 4–8).

**Table 1:** Baseline characteristics

Variable	All participants (*n* = 18)	Participants completed the study (*n* = 15)	Participants with CGM-related outcome (*n* = 11)
Age (years)	55.6 (11.6)	56.5 (12.2)	58.3 (12)
Height (cm)	165.8 (8.7)	166.2 (9.1)	168.7 (9.2)
Weight (kg)	98.6 (27.6)	98.8 (27.7)	104.7 (29)
*Sex*			
Female	15 (83%)	12 (80%)	8 (73%)
Male	3 (17%)	3 (20%)	3 (27%)
Waist circumference (cm)	107.7 (22.1)	108.3 (22.9)	113.1 (23.2)
Systolic blood pressure (mmHg)	122.6 (13.3)	124 (12)	126 (11)
Diastolic blood pressure (mmHg)	71 (8.9)	71 (10)	72 (10)
BMI (kg/m^2^)	35.7 (8.5)	35.6 (8.5)	36.7 (9.2)
HbA1c (%)	5.3 (0.5)	5.3 (0.5)	5.3 (0.5)
HbA1c (mmol/l)	34.7 (5)	34.2 (5.1)	34.7 (5.1)
*Ethnicity*			
White British	18 (100%)	15 (100%)	11 (100%)
Time from surgery (months)	78.0 (60.3)	88.8 (60.4)	86.3 (59.5)
*Bariatric surgery type*			
SG	8 (44%)	5 (34%)	3 (27%)
RYGB	10 (56%)	10 (66%)	8 (73%)

Continuous data are expressed as mean ± SD, and categorical data as count (percentage)

*SG * sleeve gastrectomy, *RYGB * Roux-Y gastric bypass, *CGM * Continuous Glucose Monitoring

### Continuous Glucose Monitoring Outcomes

Eleven participants provided complete CGM data (at least 70% data). Eight participants used a single CGM sensor across both treatment periods, whereas three participants required two separate sensors. Hypoglycaemic events were infrequent across conditions. Only one participant exhibited an isolated glucose value < 3.0 mmol/l during the monitoring period, indicating minimal exposure to clinically significant hypoglycaemia and precluding statistical analysis of this outcome. Regarding CGM variability metrics [i.e., the SD of the mean interstitial glucose and the coefficient of variation (CV)] were lower for the AEX condition compared to the CON (see Table 2, *p* < 0.01). Additionally, for nadir glucose, time spent < 3.9 mmol/l, mean 24-h glucose, no differences were observed between treatments (see Table 2 and Supplementary Table 2, *p* > 0.05). Descriptive data for the RYGB patients are reported in Supplementary Table 3.

**Table 2:** CGM outcomes

	AEX	CON	*p*-value
Time spent in interstitial glucose levels < 3.9 mmol/L (%)	0 (0.0, 0.5)	0 (0.0, 0.5)	0.68
Time spent in interstitial glucose > 10 mmol/L (%)	2.8 (1.0, 10.9)	6.6 (1.4, 12.3)	0.10
Time in interstitial glucose levels between 3.9–10 mmol/L (%)	94.8 (89.1, 97.9)	93.0 (87.3, 97.6)	0.08
Mean interstitial glucose mmol/L (mean ± SD)	6.4 (1.0)	6.5 (0.9)	0.57
Nadir interstitial glucose mmol/L (mean ± SD)	4.21 (0.78)	3.98 (0.58)	0.16
Standard deviation of the mean interstitial glucose	1.4 (1.2, 1.9)	2.0 (1.3, 2.4)	**< 0.001**
Interstitial Glucose Coefficient of variation	23.1 (20.6, 28.8)	29.8 (22.1, 32.7)	**0.004**

Data are expressed as median (quartiles), unless otherwise stated

*n = 11*

*AEX * Aerobic exercise, *CON *=Prolonged sitting

### Mixed-Meal Tolerance Test

#### Glucose and Insulin Levels

**Fig. 3:**
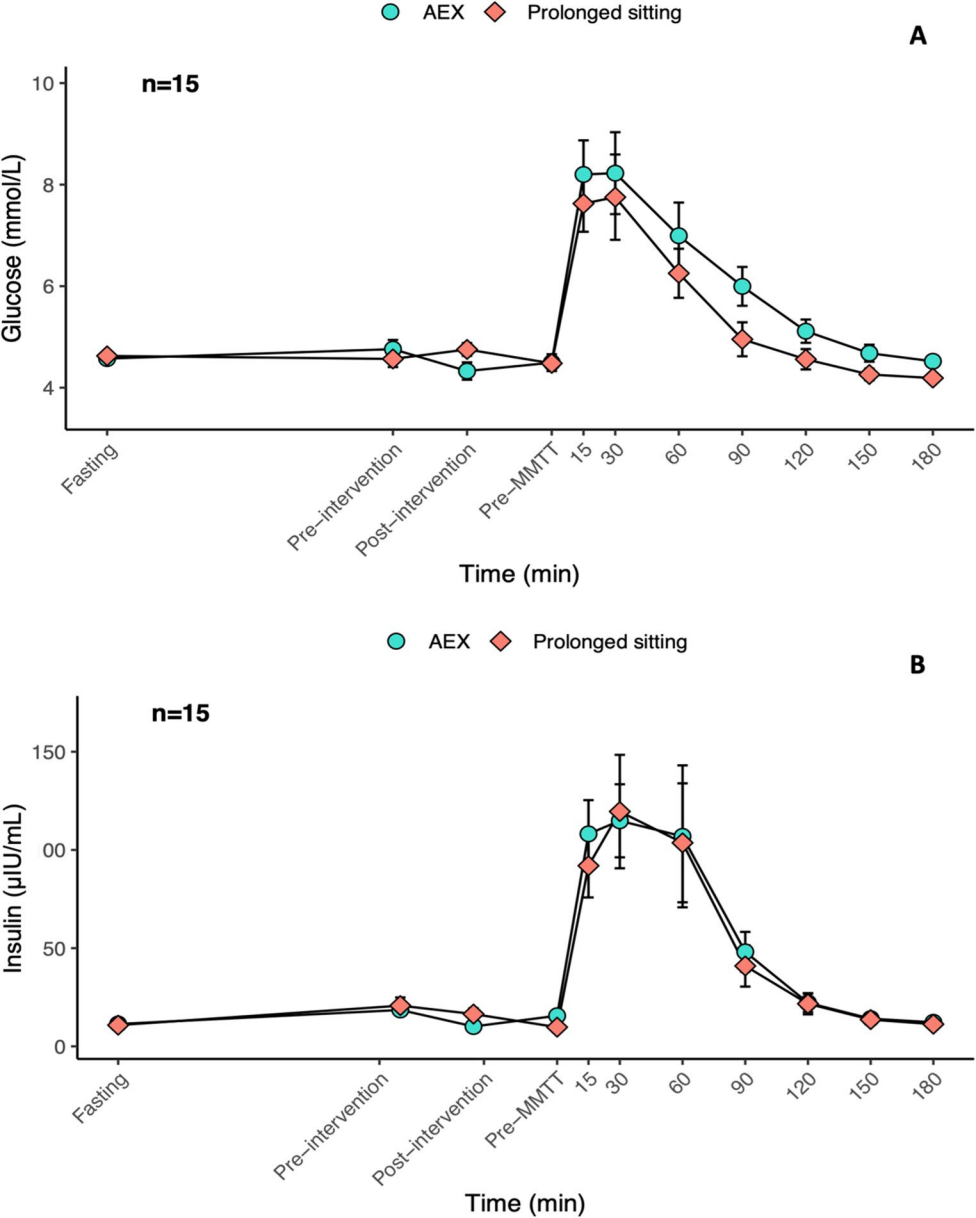
Plasma glucose (**A**) and insulin (**B**) concentrations during the intervention days

The analysis for the MMTT included fifteen participants with glucose and insulin data. For plasma glucose levels during the MMTT (see Table 3), no significant differences were observed between conditions for pre-meal glucose levels. However, the postprandial glucose nadir, peak, and the area under the curve (AUC _0−180_) were all significantly higher for the AEX condition compared to the CON condition (see Table 3; Fig. 3A). Regarding insulin levels, a significant difference was observed for pre-meal insulin levels, with the AEX condition exhibiting higher concentrations (see Table 3). In contrast, no significant differences were noted between conditions for peak and AUC _0−180_ insulin concentrations (see Table 3; Fig. 3B). Additional secondary outcomes can be found in Supplementary Table 4.

**Table 3:** Glucose and insulin responses to the MMTT

	AEX	CON	*p* value
Glucose			
Pre-meal (mmol/L)	4.49 (0.64)	4.48 (0.43)	0.20
nadir (mmol/L)	4.28 (0.72)	3.84 (0.57)	**< 0.01**
peak (mmol/L)	9.14 (2.69)	8.41 (2.61)	**0.02**
AUC _0−180_ (mmol/L x 180 min)	1269 (235)	1171 (201)	**0.02**
Insulin			
Pre-meal (µIU/mL)	15.6 (10.4)	9.76 (6.75)	**< 0.01**
Peak (µIU/mL)	159 (126)	146 (123)	0.21
AUC _0−180_ (µIU/mL x 180 min)	10 760 (7915)	10 231 (8391)	0.05

Data are expressed as mean ± SD. Models were adjusted accordingly for the intervention group, visit, fasting, pre-exercise, and post-exercise glucose or insulin values

*n* = 15

*AEX* Aerobic exercise, *CON* Prolonged sitting, *AUC* Area Under the Curve

When analysing the RYGB participants (refer to Supplementary Table 5), the results were consistent with those presented here. Notably, in this subgroup, both pre-meal glucose and insulin AUC_0−180_ reached also statistical significance, with higher pre-meal glucose and insulin concentrations for AEX group. Due to the small sample size, a similar analysis was not conducted for the SG participants. Graphical representations for both RYGB and SG participants are available in the supplementary material for glucose and insulin (Supplementary Figs. 1 & 2).

Pre-MMTT represents the baseline sample taken before the MMTT. Subsequent sample times (15, 30, 60, 90, 120, 150 and 180 min) are expressed as time elapsed since meal consumption was completed. Data are presented as mean ± standard errors. Circles denote the AEX condition, and rhomboids denote the CON condition. AEX = Moderate-intensity Aerobic Exercise; MMTT = Mixed-Meal Tolerance Test.

#### Dietary Intake

Of the 15 participants who completed the study, one did not provide one or both required food diary records and was excluded from the analysis. As a result, 14 participants were included in this analysis. No significant differences in total energy, carbohydrates, protein, and fat intake within the 24 h following the intervention across both treatment periods were observed (*p* > 0.05) (see Supplementary Table 6).

#### Physical Activity

No differences between conditions were observed for MVPA for the 24-h pre- (AEX: 6.6 ± 6.7 vs. CON: 7.4 ± 6.8 min; *p* = 0.62; *n* = 8) and post-intervention (AEX: 8.0 ± 6.4 vs. CON: 6.9 ± 9.7 min; *p* = 0.54; *n* = 10).

#### Safety Data

Supplementary Table 7 summarises all adverse events (AEs). Twenty-six events were reported in 10/18 participants (56%), with no serious adverse events (SAEs). Eleven occurred before to the first treatment period, 8 during AEX and 7 during CON; headache was the most frequently reported event.

## Discussion

The present study examined the impact of a 30-min session of AEX before lunch on CGM glucose levels in adult participants without a diagnosis of PHH who had undergone MBS at least 12 months prior. The results showed that AEX did not significantly affect CGM time spent in hypoglycaemia (interstitial glucose < 3.0 mmol/L) or nadir glucose concentrations over the following 24 h. However, AEX resulted in reduced glycaemic variability over 24 h compared to the prolonged sitting group (CON). In addition, the AEX condition resulted in higher postprandial glucose levels after the MMTT (lunch) compared to the CON condition. This included a higher glucose nadir, peak, and AUC_180_, along with elevated insulin concentrations before the MMTT. Overall, a single pre-lunch AEX session was associated with a more stable 24-h glycaemic profile and, elevated postprandial glucose levels and glucose nadir after a MMTT, suggesting no increased risk of PHH after MBS.

MBS has been linked to increased risk of PHH and increased amount of time spent in hypoglycaemia [29, 30]. However, in the present study, neither experimental condition led to significant time spent in hypoglycaemia. This contrasts with previous observations from CGM, which reported that individuals typically spend 2–5% of their time with glucose levels below 3.0 mmol/L in daily life [3, 7]. One possible explanation for this discrepancy is the controlled nature of the study, where participants adhered to a standardised diet consumed at specific time points. Participants’ mean energy intake over the 24 h post-intervention was 5,439 kJ/day (50% carbohydrates, 35% fat, 15% protein), slightly below the typical first-year postoperative intake of ~ 7,120 kJ/day [31, 32]. The observed difference likely results from the exclusion of snacks post-intervention. Nevertheless, PHH is mainly induced by main meals high in carbohydrates (> 30 g/meal) [2], while snacks appear to contribute minimally. Furthermore, to ensure participant safety, individuals with a known diagnosis of PHH were excluded from the study.

Patients who have undergone MBS, particularly RYGB, generally show greater glycaemic variability than healthy controls [33–35], which is associated with an increased risk of PHH [36]. In the present study, the mean glucose standard deviation and coefficient of variation were significantly greater in the CON than in the AEX condition, suggesting that a single bout of AEX may lower glycaemic variability after MBS. This finding aligns with previous research demonstrating the beneficial effects of acute AEX on glycaemic variability in both healthy individuals and those with chronic conditions [37, 38]. To our knowledge, our study is the first to assess the impact of a single AEX session on CGM-derived glucose variability and other metrics in individuals who have undergone MBS. The study’s hybrid design (combining supervised lab-based exercise and meals with at-home standardized meals and CGM), enhances the real-world relevance of our findings.

During the MMTT (lunch), the AEX condition revealed a distinct glycaemic pattern. Participants in the AEX condition exhibited higher nadir, peak, and total AUC (0–180 min) blood glucose concentrations during the MMTT, suggesting increased postprandial glucose levels in the immediate post-lunch period. Consistent with our findings, previous studies in healthy individuals who have not undergone MBS have shown that performing AEX prior to a second meal can increase postprandial glucose levels by approximately 15% [39]. This so-called paradoxical post-exercise second-meal phenomenon may be explained by increased intestinal glucose absorption, elevated hepatic glucose production following exercise (potentially influenced by increased secretion of catecholamines and glucagon), and reduced glucose uptake, particularly in inactive or exercise-fatigued muscles [39]. Furthermore, mechanistic studies in dogs have shown that elevated levels of glucagon and catecholamines early in the day, such as those possibly triggered by AEX, can increase endogenous glucose production without a corresponding rise in peripheral glucose uptake [40], thereby raising blood glucose concentrations.

In our study, postprandial insulin concentrations did not differ between conditions, suggesting that differences in circulating insulin were unlikely to be the principal explanation for the elevated glucose response. However, insulin concentrations were higher immediately before the second meal in the AEX condition. This finding is more likely to reflect acute physiological responses associated with the exercise bout than differences in postprandial insulin secretion itself. Acute exercise can alter the hormonal milieu by increasing counter-regulatory factors such as glucagon and catecholamines, which may promote hepatic glucose output and transiently reduce insulin sensitivity. These responses could contribute to higher circulating glucose concentrations and may also influence circulating insulin concentrations through changes in secretion and/or clearance. Although these hormones were not measured in the present study, such exercise-related endocrine responses may partly account for the higher pre-meal insulin concentrations observed in the AEX condition; however, further work is needed to directly test these mechanisms.

The nutritional context of the protocol also distinguishes the present study from most previous second-meal research. Participants consumed a standardised carbohydrate snack 15 min before the intervention in both conditions, meaning that the pre-lunch period occurred in the context of a recent nutritional stimulus. This differs from earlier studies in which no additional snack was provided, and exercise was performed solely after breakfast [39]. Nevertheless, because the snack was standardised and identical across both trial arms within a crossover design, it is less likely to be the main explanation for the between-condition differences observed, although it does limit direct comparability with prior work. Thus, while the snack may have influenced the broader metabolic background, the higher pre-meal insulin concentrations observed in the exercise condition were likely driven by the exercise bout itself.

A previous randomised crossover study conducted in individuals without diabetes after RYGB also found that 30 min of AEX (at 70% V̇O_2_ max) performed shortly (10 min) after either a high- or low-glycaemic index breakfast did not increase hypoglycaemia risk for the next 4 h in people with and without symptoms suggestive of PHH. In fact, there was a trend toward higher nadir glucose levels compared to the resting condition (26). However, the same study observed an immediate decline in glucose levels following breakfast once AEX began. This suggests that both the timing of exercise and the type of meal consumed may influence metabolic responses. In our study, the AEX was carried out approximately 40 min before the second meal of the day (lunch) and immediately following a snack, with postprandial glucose measured after lunch. This timing may have influenced postprandial glucose dynamics through distinct physiological mechanisms compared to exercise performed after breakfast.

The glycaemic responses after AEX build upon the already altered prandial glucose regulation seen in individuals post-MBS, which includes increased post-meal glucagon secretion, enhanced insulin release, and faster gastric emptying [41–45]. While we observed higher postprandial glucose concentrations during the MMTT after pre-lunch AEX, CGM data over the subsequent 24 h showed no differences in average glucose concentrations or time spent above 10 mmol/L. This indicates that the AEX post-lunch glucose rise is likely transient (0–4 h), with no sustained elevation throughout the day. These findings support the safety of performing AEX before lunch in individuals without known PHH, as it does not appear to increase the risk of hypoglycaemia within a 24-h period. If a similar glycaemic response to pre-lunch AEX is observed in individuals with established PHH after MBS, this exercise timing may offer a potential therapeutic strategy to reduce post-lunch hypoglycaemia risk. However, studies in individuals with diagnosed PHH are required to confirm this.

While this study offers valuable insights, several limitations should be considered. The small sample size (composed predominantly of White British females who had undergone RYGB) limits the generalisability of the findings to other populations and bariatric procedures. The present study was not powered to evaluate procedure-specific effects of AEX on postprandial glucose homeostasis or to compare responses between RYGB and SG. This is particularly relevant as RYGB and SG may influence postprandial glucose regulation and incretin responses to different extents. Larger, adequately powered studies are therefore needed to determine whether exercise affects glucose homeostasis differently across procedures. Even so, the use of a controlled diet and detailed physical activity monitoring supports the study’s internal validity. Moreover, the hybrid study’s design, which included supervised lab-based components alongside at-home standardised meals, introduced some real-world variability while preserving experimental control. Although glucose and insulin measurements were the main focus of the MMTT assessments, other important regulators of glucose homeostasis such as glucagon, glucagon-like peptide-1 (GLP-1), and catecholamines were not assessed.

Additionally, the study examined only the acute effects of a single, pre-lunch session of moderate-intensity AEX in individuals without diagnosed PHH who were not regularly participating in structured exercise, as people with PHH were excluded for safety reasons. It remains unclear whether similar responses would occur with repeated exercise, in physically active individuals, or in those with PHH. Because participants were deliberately recruited without known PHH, the findings are not generalisable to individuals with established PHH, and the low number of hypoglycaemic events in this study should be interpreted in this context. Future research should consider a broader range of exercise intensities, durations, and frequencies, and investigate the role of 24-h movement behaviours - including light activity, sedentary time, and sleep - in shaping postprandial metabolic regulation after MBS. Despite these considerations, the study offers clinically relevant insights under conditions that approximate real-world settings, supporting the short-term safety of pre-lunch AEX in the post-MBS population.

In summary, the present study found that performing 30 min of moderate-intensity AEX before lunch (and immediately after a snack), in individuals ≥ 12 months post-MBS, did not increase the time spent in hypoglycaemia over the subsequent 24 h period. Instead, this AEX session resulted in a rise in blood glucose concentrations during the immediate post-lunch period. These findings indicate that incorporating pre-lunch AEX into daily routines may be safe and does not appear to increase the risk of PHH. However, further studies are needed to determine whether these outcomes apply to other populations, such as individuals with PHH after MBS, and to explore the effects of different exercise prescriptions, timing, and prandial states.

## Supplementary Information

Below is the link to the electronic supplementary material.

Supplementary Material 1 (DOCX 486 KB)

## Data Availability

Data included in this manuscript is available upon request from the corresponding author.
