# Supplementary material for: The Effect of Acute Aerobic Exercise On the Time Spent in Hypoglycaemia After Bariatric Surgery (The BariEX Study)
Source: Obes Surg. 2026 May 1;36(6):3058–70. doi: 10.1007/s11695-026-08712-3 (PMC13249763; doi:10.1007/s11695-026-08712-3)
Supplement: Supplementary file 1 — Supplementary Material 1 (DOCX 486 KB) [file 11695_2026_8712_MOESM1_ESM.docx]

**The effect of acute aerobic exercise on the time spent in hypoglycaemia after bariatric surgery (The BariEX Study)**

**Supplementary Table 1. Baseline characteristics for RYGB subgroup analysis.**

| Variable | RYGB subgroup analysis  (n=10) |
| --- | --- |
| Age (years) | 61.4 (10.8) |
| Height (cm) | 167.1 (10.1) |
| Weight (kg) | 101.3 (28) |
| *Sex* |  |
| Female | 7 (70%) |
| Male | 3 (30%) |
| Waist circumference (cm) | 113.6 (20.8) |
| Systolic blood pressure (mmHg) | 126 (11) |
| Diastolic blood pressure (mmHg) | 74 (8) |
| BMI (kg/m^2^) | 36.1 (8.3) |
| HbA1C (%) | 5.6 (0.4) |
| HbA1C (mmol/L) | 36.5 (4.6) |
| *Ethnicity* |  |
| White British | 10 (100%) |

Continuous data are expressed as mean ± SD, and categorical data as count (percentage). RYGB = Roux-Y gastric bypass.

**Supplementary Table 2. Other continuous glucose monitoring (CGM) secondary outcomes (n=11).**

|  | **AEX** | **CON** | p-value |  |
| --- | --- | --- | --- | --- |
| **Time spent in interstitial glucose >7.8 mmol/L (%)** | 9.4 (8.3, 20.8) | 13.7 (11.5, 21.0) | 0.28 |  |
| **Time spent in interstitial glucose levels between 3.0 – 7.8 mmol/L (%)** | 90.6 (79.2, 91.3) | 86.3 (79.0, 88.5) | 0.31 |  |
| **Time spent in interstitial glucose levels between 3.9 – 7.8 mmol/L (%)** | 88.9 (79.2, 90.6) | 85.4 (78.7, 88.4) | 0.31 |  |
| **Risk of hypoglycaemia calculated as the low blood glucose index** | 274.9 | 273.8 | 0.79 |  |

Data are expressed as median and quartiles, unless otherwise stated. AEX = Aerobic exercise; CON = Prolonged sitting. n =11.

**Supplementary Table 3. Descriptive continuous glucose monitoring (CGM) outcomes for the RYGB subgroup.**

|  | **AEX** | **CON** |
| --- | --- | --- |
| **Time spent in interstitial glucose >10 mmol/L (%)** | 7.46 (2.78, 12.40) | 10.59 (5.56, 13.94) |
| **Time in interstitial glucose levels between 3.9 – 10 mmol/L (%)** | 92.36 (87.76, 95.41) | 89.41 (86.29, 94.18) |
| **Mean interstitial glucose mmol/L (mean ± SD)** | 6.53 (6.09, 7.58) | 6.89 (6.17, 7.12) |
| **Nadir interstitial glucose mmol/L (mean ± SD)** | 4.4 (0.82) | 4.16 (0.56) |
| **Standard deviation of the mean interstitial glucose** | 1.82 (1.37, 1.97) | 2.19 (1.81, 2.41) |
| **Interstitial Glucose Coefficient of variation** | 25.93 (21.42, 30.10) | 30.79 (27.78, 33.82) |
| **Time spent in interstitial glucose >7.8 mmol/L (%)** | 15.60 (9.11, 21.96) | 17.71 (11.63, 21.35) |
| **Time spent in interstitial glucose levels between 3.0 – 7.8 mmol/L (%)** | 84.40 (78.04, 90.71) | 82.29 (78.64, 88.37) |
| **Time spent in interstitial glucose levels between 3.9 – 7.8 mmol/L (%)** | 84.40 (78.04, 89.32) | 82.29 (78.13, 88.11) |
| **Risk of hypoglycaemia calculated as the low blood glucose index** | 268.75  (246.10, 283.03) | 264.41  (256.45, 280.78) |

Data are expressed as median (quartiles), unless otherwise stated. AEX = Aerobic exercise; CON = Prolonged sitting; RYGB = Roux-Y gastric bypass. n =8.

**Supplementary Table 4. Secondary outcomes for glucose and insulin responses to the MMTT**

|  | **AEX** | **CON** | p value |
| --- | --- | --- | --- |
| Ratio of maximum/minimum plasma glucose | 2.17 (0.65) | 2.21 (0.66) | 0.78 |
| Ratio AUC_0-180_ insulin/AUC_0-180_ glucose | 5.27 (4.11, 11.80) | 5.64 (3.81, 11.53) | 0.98 |
| Ratio AUC_0-30_ insulin/AUC_0-30_ glucose | 5.65 (4.62, 11.25) | 4.24 (3.26, 11.47) | 0.25 |
| Ratio AUC_60-180_ insulin/AUC_60-180_ glucose | 3.32 (2.65, 9.75) | 4.32 (2.35, 9.68) | 0.93 |

Data are expressed as mean ± SD or median (quartiles). Models were adjusted accordingly for the randomisation group, visit, fasting, and pre-exercise and post-exercise glucose or insulin values. For the AUC insulin/AUC glucose ratios, Wilcoxon signed-rank tests were applied. AEX = Aerobic exercise; CON = Prolonged sitting, AUC: Area under the curve. n =15.

**Supplementary Table 5. Primary and secondary outcomes for glucose and insulin responses to the MMTT for the RYGB subgroup (n=10).**

|  | **AEX** | **CON** | p value |
| --- | --- | --- | --- |
| **Glucose** |  |  |  |
| Pre-meal (mmol/L) | 4.77 (0.59) | 4.55 (0.44) | **0.04** |
| nadir (mmol/L) | 4.28 (0.80) | 3.85 (0.69) | **0.04** |
| peak (mmol/L) | 10.4 (2.30) | 9.48 (2.54) | **0.02** |
| AUC _0-180_ (mmol/L x 180 min) | 1341 (233) | 1224 (237) | **0.01** |
| Ratio of maximum/minimum plasma glucose | 2.48 (0.56) | 2.50 (0.63) | 0.98 |
| **insulin** |  |  |  |
| Pre-meal (µIU/mL) | 16.1 (11.4) | 9.73 (6.82) | **0.01** |
| Peak (µIU/mL) | 184 (143) | 182 (132) | 0.32 |
| AUC _0-180_ (µIU/mL x 180 min) | 12275 (8532) | 12256 (9309) | **0.04** |
|  |  |  |  |
| **Ratio AUC_0-180_ insulin/AUC_0-180_ glucose** | 5.74 (4.95, 13.15) | 6.23 (4.26, 14.79) | 0.32 |
| **Ratio AUC_0-30_ insulin/AUC_0-30_ glucose** | 6.91 (4.89, 9.22) | 7.31 (3.82, 11.67) | 0.92 |
| **Ratio AUC_60-180_ insulin/AUC_60-180_ glucose** | 3.78 (2.77, 10.18) | 5.72 (2.75, 10.41) | 1.00 |

Data are expressed as mean ± SD or median (quartiles). Models were adjusted accordingly for the randomisation group, visit, fasting, and pre-exercise and post-exercise glucose or insulin values. For the AUC insulin/AUC glucose ratios, Wilcoxon signed-rank tests were applied. AEX = Aerobic exercise; CON = Prolonged sitting, AUC: Area under the curve. n =10.

**Supplementary Figure 1. Plasma glucose concentrations for the RYGB (top) and SG (bottom) patients during the intervention days.** Pre-MMTT represents the baseline sample taken before the MMTT. Subsequent sample times (15,30,60,90,120,150 and 180 min) are expressed as time elapsed since meal consumption completed. Data are presented as mean ± standard errors. Circles denote the AEX condition, and rhomboids denote the CON condition. RYGB = Roux-Y gastric bypass; SG = Sleeve gastrectomy.

**
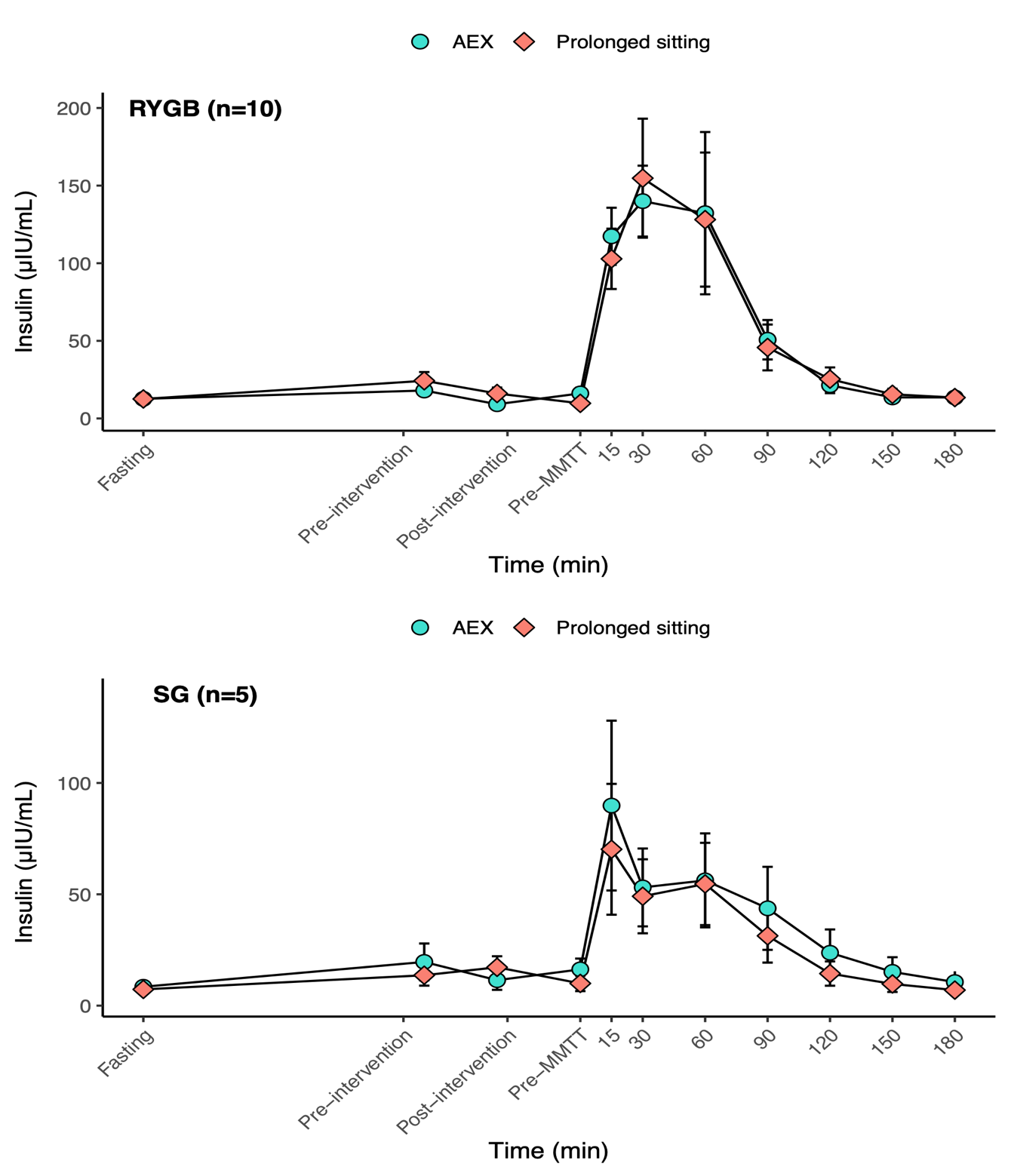
**

**Supplementary Figure 2. Plasma insulin concentrations for the RYGB (top) and SG (bottom) patients during the intervention days.** Pre-MMTT represents the baseline sample taken before the MMTT. Subsequent sample times (15,30,60,90,120,150 and 180 min) are expressed as time elapsed since meal consumption completed. Data are presented as mean ± standard errors. Circles denote the AEX condition, and rhomboids denote the CON condition. RYGB = Roux-Y gastric bypass; SG = Sleeve gastrectomy.

**Supplementary Table 6:** **Participants’ dietary intake during the 24 h following the interventions.**

|  | **AEX** | **CON** | *p-value* |
| --- | --- | --- | --- |
| **Dietary variables** |  |  |  |
| Energy (kcal/day) | 1271.3 ± 295.3 | 1349.2 ± 299.5 | 0.13 |
| Carbohydrates (g/day) | 151.4 ± 37.5 | 159.1 ± 37.6 | 0.36 |
| Protein (g/day) | 55.3 ± 12.6 | 54.5 ± 9.6 | 0.72 |
| Fat (g/day) | 53.5 ± 12.2 | 54.9 ± 14.0 | 0.60 |

Data are expressed as mean ± SD. AEX = Aerobic exercise; CON = Prolonged Sitting. n =14.

**Supplementary Table 7: Safety data**

|  | **AEX** | | **CON** | | **Before initiation of 1^st^ treatment period** | | **Total** | |
| --- | --- | --- | --- | --- | --- | --- | --- | --- |
|  | Patients | Events | Patients | Events | Patients | Events | Patients | Events |
| SAEs | 0/18 (0%) | 0 | 0/18 | 0 | 0/18 | 0 | 0/18 | 0 |
| Any AEs | 7/18 (39%) | 8 | 4/18 (22%) | 7 | 6/18 (33%) | 11 | 10/18 (56%) | 26 |
| **Nervous system disorders** |  |  |  |  |  |  |  |  |
| Headache | 3/18 (17%) | 3 | 3/18 (17%) | 3 | 3/18 (17%) | 3 | 7/18 (39%) | 9 |
| Dizziness | 0/18 (0%) | 0 | 1/18 (6%) | 0 | 0/18 (0%) | 0 | 1/18 (6%) | 1 |
| Off balance | 0/18 (0%) | 0 | 1/18 (6%) | 0 | 0/18 (0%) | 0 | 1/18 (6%) | 1 |
| **Metabolism and nutrition disorders** |  |  |  |  |  |  |  |  |
| Hypoglycaemia | 0/18 (0%) | 0 | 1/18 (6%) | 1 | 3/18 (17%) | 5 | 3/18 (17%) | 6 |
| **Infections and infestations** |  |  |  |  |  |  |  |  |
| Influenza | 0/18 (0%) | 0 | 0/18 (0%) | 0 | 1/18 (6%) | 1 | 1/18 (6%) | 1 |
| Head cold | 0/18 (0%) | 0 | 0/18 (0%) | 0 | 1/18 (6%) | 1 | 1/18 (6%) | 1 |
| Cold | 1/18 (6%) | 1 | 0/18 (0%) | 0 | 0/18 (0%) | 0 | 1/18 (6%) | 1 |
| **Skin and subcutaneous tissue disorders** |  |  |  |  |  |  |  |  |
| Itchy rash | 1/18 (6%) | 1 | 0/18 (0%) | 0 | 0/18 (0%) | 0 | 1/18 (6%) | 1 |
| Clamminess | 1/18 (6%) | 1 | 0/18 (0%) | 0 | 0/18 (0%) | 0 | 1/18 (6%) | 1 |
| **Eye disorders** |  |  |  |  |  |  |  |  |
| Eye discomfort | 1/18 (6%) | 1 | 0/18 (0%) | 0 | 0/18 (0%) | 0 | 1/18 (6%) | 1 |
| **Blood and lymphatic system disorders** |  |  |  |  |  |  |  |  |
| Iron deficiency | 0/18 (0%) | 0 | 0/18 (0%) | 0 | 1/18 (6%) | 1 | 1/18 (6%) | 1 |
| **Gastrointestinal disorders** |  |  |  |  |  |  |  |  |
| Nausea | 0/18 (0%) | 0 | 1/18 (6%) | 0 | 0/18 (0%) | 0 | 1/18 (6%) | 1 |
| **Injury, poisoning and procedural complications** |  |  |  |  |  |  |  |  |
| Finger fracture | 1/18 (6%) | 1 | 0/18 (0%) | 0 | 0/18 (0%) | 0 | 1/18 (6%) | 1 |

Abbreviations: AEX: Aerobic exercise, CON: Control condition, AE: Adverse Event, SAE: Serious Adverse Event.

**Inclusion and exclusion criteria**

Eligible participants included those who had undergone MBS (RYGB or SG) at least ≥12 months before entering the trial, aged ≥18 to <75 years, without diabetes, able to understand written and spoken English and able to provide informed consent. Participants were excluded from the study if they met any of the following criteria: current type 2 diabetes (defined as HbA1C ≥6.5% at screening, or HbA1C <6.5% but receiving glucose-lowering medications within the past 3 months); type 1 diabetes; eGFR ≤30 ml/min at screening; history of revisional bariatric surgery (except previous gastric banding subsequently removed); bariatric procedures other than RYGB or SG (single anastomosis gastric bypass was excluded); established diagnosis of PHH; use of medications known to affect glucose levels (e.g., glucose-lowering medications, systemic corticosteroids [excluding topical or inhaled formulations], acarbose, diazoxide, or octreotide); contraindications or limitations to aerobic exercise (self-reported or identified during maximal exercise testing, e.g., orthopaedic limitations, severe cardiovascular/pulmonary disease, being chair-bound); uncontrolled hypertension (systolic blood pressure ≥160 mmHg or diastolic blood pressure ≥100 mmHg at screening); regular participation in structured exercise (as defined by the International Physical Activity Questionnaire Short Form (IPAQ-SF)); history of epilepsy; haemoglobin <100 g/L at screening; current pregnancy or breastfeeding; recent active infection (within the previous 10 days); adrenal insufficiency and/or ongoing glucocorticoid substitution therapy; body weight >180 kg due to treadmill capacity limits; participation in another intervention-based research study within the 3 months prior to screening; or severe intolerance to the standardised meals, as determined by a clinician during the screening visit.

**Intervention**

In both conditions, participants remained under the supervision of a trained staff member for the entire 35-min session. In the AEX condition, they began with a 3-min warm-up, then completed a 30-min brisk treadmill walk at the speed and incline required to elicit 60 % of their V̇O₂ peak and finished with a 2-min cool-down. In the CON condition, participants remained resting and seated throughout the same 35-min period. Blood pressure was measured immediately before and just after the AEX session. Additionally, heart rate and the rating of perceived exertion (RPE) were continuously recorded throughout the AEX condition.

**Maximal exercise test protocol**

A graded treadmill maximal exercise test was undertaken at a fixed speed with progressively increasing gradient. The test was used to ensure participants’ safety and to determine their maximum aerobic capacity (*V̇*O_2_peak), informing the exercise intensity. The test included participants’ pre-screening, involving medical history review and resting electrocardiography (ECG).

After familiarisation with the treadmill, participants completed a 5-minute warm-up (speed ≤ 4km/h). Then participants walked at a fixed speed at a 0% gradient before the gradient increased by 1% each minute for the test duration. RPE (6-20), heart rate (Polar chest strap, Polar, Finland) and expired air (CORTEX Metalyzer 3B, CORTEX Biophysik GmbH, Leipzig, Germany) were collected during the test. Participants were encouraged to continue the test until (a) volitional exhaustion, (b) participants reached 100% of their age-predicted maximum heart rate (85% if taking β-blockers) and a Respiratory Exchange Ratio (RER) of ≥ 1.15 or (c) the test was terminated by the cardiac nurse/clinician, who was monitoring a 12-lead ECG. The latter was classified as an incomplete test, and participants were referred for further investigation if needed. A rolling average of 10 breaths was calculated for *V̇*O_2_ and *V̇*CO_2,_ and peak oxygen uptake (*V̇*O_2peak_) was defined as the highest value.

**Dietary data ­**

**Food provision–Standardised meals**

Standardised meals were provided for the evening meal on day 1, breakfast, lunch and evening meal on day 2, and breakfast and lunch on day 3. Food preferences were obtained from a pre-determined menu. Reasonable adjustments were made for participants (n=3) with dietary restrictions (food intolerance). Participants’ individual total daily energy intake requirements were calculated using the Mifflin-St Jeor equation, which used their age, height, weight and physical activity level to estimate their total daily energy expenditure. On the evening of day 1 and 2, the provided standardised meal contained approximately 2050 kJ (15% protein, 47% carbohydrates, 38% fat). During day 2, breakfast (consumed on site) provided approximately 15% of estimated daily energy requirements (13% protein, 53% carbohydrates, 34% fat). A standardised 15-gram carbohydrate snack (452 kJ, 6% protein, 56% carbohydrates, 38% fat) was provided 15 min before the exercise to help avoid PHH during the exercise, and at the same timing during the CON condition. The MMTT on day 2 was provided as lunch and is described in detail at the next section (mixed meal tolerance test). The breakfast on day 3 contained approximately 1259 kJ (17% protein, 47% carbohydrates, 36% fat). When required by participants, adapted meals were provided with similar caloric and macronutrient profiles (for gluten free or lactose free requirements). The total average macronutrient composition of standardised meals provided over the course of the study followed the dietary reference values for the UK population [protein (15%), carbohydrate (50%) and fat (35%)]. The meals consumed at the first treatment period were replicated at the second treatment period.

***Physical Activity - Accelerometer***

Participants wore a GENEActiv accelerometer (ActivInsights, Kimbolton, UK) on their non-dominant wrist to quantify habitual MVPA levels from visit 0 throughout the study duration, specifically from visit 0 to visit 5. The devices were configured to record data at a frequency of 20 Hz over a period of 30 days. The raw data processing was conducted using the open-source GGIR package (version 3.1-5) in R (1) to determine time spent in MVPA (> 100 m*g*) (2), in min accumulated in ≥ 1-min bouts (3). These data were utilised to evaluate any differences in MVPA in the 24-h period before and after the intervention. Unfortunately, for technical reasons (unexpected battery depletion in some devices), only eight and ten participants were included in the comparison of pre- and post-intervention MVPA levels, respectively.

**References:**

1. Migueles JH, Rowlands A V., Huber F, Sabia S, Van Hees VT. GGIR: A Research Community–Driven Open Source R Package for Generating Physical Activity and Sleep Outcomes From Multi-Day Raw Accelerometer Data. J Meas Phys Behav. 2019 Sep 1;2(3):188–96. doi:10.1123/JMPB.2018-0063

2. Hildebrand M, Van Hees VT, Hansen BH, Ekelund U. Age group comparability of raw accelerometer output from wrist-and hip-worn monitors. Med Sci Sports Exerc. 2014;46(9):1816–24. doi:10.1249/MSS.0000000000000289, PubMed PMID: 24887173.

3. Menai M, Van Hees VT, Elbaz A, Kivimaki M, Singh-Manoux A, Sabia S. Accelerometer assessed moderate-To-vigorous physical activity and successful ageing: Results from the Whitehall II study. Sci Rep. 2017 Apr 3;8(1):1–9. doi:10.1038/SREP45772;SUBJMETA=174,308,499,692;KWRD=EPIDEMIOLOGY,RISK+FACTORS PubMed PMID: 28367987.
